# Circadian Timing, Information processing and Metabolism (TIME) study: protocol of a longitudinal study of sleep duration, circadian alignment and cardiometabolic health among overweight adults

**DOI:** 10.1186/s12902-023-01272-y

**Published:** 2023-01-31

**Authors:** Kelly Glazer Baron, Bradley M. Appelhans, Helen J. Burgess, Lauretta Quinn, Tom Greene, Chelsea M. Allen

**Affiliations:** 1 Division of Public Health, Department of Family and Preventive Medicine, University of Utah, 375 Chipeta Way, Suite A, UT Salt Lake City, 84108 US; 2 Department of Family and Preventive Medicine, Rush University Medical Center, Chicago, IL US; 3 Department of Psychiatry, University of Michigan, Ann Arbor, MI US; 4 School of Nursing, University of Illinois at Chicago, Chicago, IL US; 5 Department of Population Health Sciences, University of Utah, Salt Lake City, UT US

**Keywords:** Obesity, Metabolism, Circadian, Sleep, Diet

## Abstract

**Background:**

Both short sleep duration and circadian rhythm misalignment are risk factors for metabolic dysfunction, but the underlying mechanisms are unknown. The goal of this study is to examine how sleep duration and circadian alignment predict changes in cardiometabolic risk factors over a 12-month period, and test cognitive function and hedonic eating tendencies as potential mechanisms.

**Methods:**

We will recruit a sample of 120 working aged adults with BMI 25–35 kg/m^2^ (overweight to class I obesity). The protocol includes 5 visits over a 12-month period. Study visits include wrist actigraphy to measure sleep behaviors, 24-h diet recalls, dim light melatonin collection, a computerized neurobehavioral assessment, eating in the absence of hunger task, and frequently sampled IV glucose tolerance test.

**Discussion:**

The results of the TIME study will advance the understanding of how both short sleep duration and circadian misalignment contribute to behavioral aspects of obesity and metabolic dysfunction.

**Trial registration:**

ClinicalTrials.Gov, NCT04759755, registered retrospectively February 13, 2021.

## Background

More than 1/3^rd^ of adults are overweight or obese [1] which contributes to major causes of preventable death including cardiometabolic diseases (cardiovascular disease, type 2 diabetes) as well as some cancers. There is increasing evidence that both short sleep duration and circadian rhythm misalignment adversely impact cardiometabolic health [2]. A meta-analysis demonstrated short sleep duration was associated with a 37% higher risk for diabetes mellitus, 16% higher risk for cardiovascular disease and 38% higher risk for obesity [3]. In addition, circadian misalignment, defined as mistimed sleep in relation to internal circadian rhythm, is also an independent predictor of cardiometabolic health [4, 5]. When exposures to short sleep duration and circadian misalignment are combined in laboratory experiments, research has demonstrated these exposures have an additive effect [6, 7]. However, little is known about how chronic exposure to sleep loss and circadian misalignment in daily life affect cardiometabolic health.

Our laboratory has focused on the translation of laboratory research to real-world settings and studying the effects of common types of circadian misalignment in daily life. In our previous study, we examined the role of circadian timing and alignment (sleep timing in relation to the circadian phase marker of dim light melatonin onset) on obesity and cardiometabolic risk factors in a sample of healthy young to middle aged adults with habitual sleep duration of at least 6.5 h [8]. We discovered that circadian misalignment, rather than timing per se, was associated with greater caloric and carbohydrate intake, consuming a greater number of meals, and in overweight participants, insulin resistance [8, 9].

A limitation of our study design was that by excluding individuals with short sleep duration, we could not examine the role of sleep duration in this study. Given that more than half of potential participants were ineligible for our study due to short sleep duration (particularly those with later sleep times), this demonstrates how the interaction between sleep duration and circadian alignment may be particularly important to understanding risk in daily life. In addition, results of our study also suggest that neurobehavioral determinants of increased hedonic eating (e.g., impulsivity) may play a role in the cardiometabolic consequences of short sleep duration and circadian misalignment.

This paper describes the protocol of our current study: the circadian Timing, Information processing and Metabolism (TIME) study. The first aim of the study is to examine the interaction between sleep duration and circadian misalignment in predicting dietary behavior, neurobehavioral aspects of hedonic eating behaviors, dietary intake and cardiometabolic risk factors. The study also will examine neurobehavioral measures in the morning versus the evening and finally evaluate how sleep and circadian misalignment predict 12-month change in cardiometabolic risk factors. Recruitment for this study began in 2019, therefore we also describe the protocol adaptions we made due to the COVID-19 pandemic.

## Methods/Design

### Participants and eligibility criteria

Participants will include adults aged 18–55 years with BMI ranging from 25.0–34.9 kg/m^2^who have habitual sleep onset times between 10:30 pm and 3:00 am, verified by 7 days of wrist actigraphy. Exclusionary criteria include: 1) High risk or presence of sleep disorders as assessed by the questionnaires and overnight OSA screening; 2) Diabetes diagnosis or HbA1c (glycated hemoglobin) > 7.0%; 3) History of cognitive or neurological disorders; 4) Presence of major psychiatric disorders, current alcohol or substance abuse as determined by screening questionnaires; 5) Unstable or serious medical illness; 6) Overnight shift work or travel over 2 or more time zones in the past 2 months; 7) Use of hypnotic, stimulant or medications known to affect melatonin concentrations such as beta blockers, daily NSAIDs or antidepressants; 8) Current smoking, 9) Daily caffeine intake > 300 mg, 10) Pregnant or lactating.

### Procedure

#### Recruitment and prescreening

Participants will be recruited from the community using flyers, mailing letters, research registries (researchmatch.org) and online postings (Reddit, Craig’s list, Facebook). Interested participants will complete screening questionnaires online via the REDCap system and then study staff will follow-up with phone calls to review key criteria, administer the substance abuse and eating disorders questionnaires and discuss information about the study. If interested and eligible, participants will be scheduled for an in-person study visit and tentatively scheduled for the follow-up visits.

#### Study visits

**Fig. 1:**
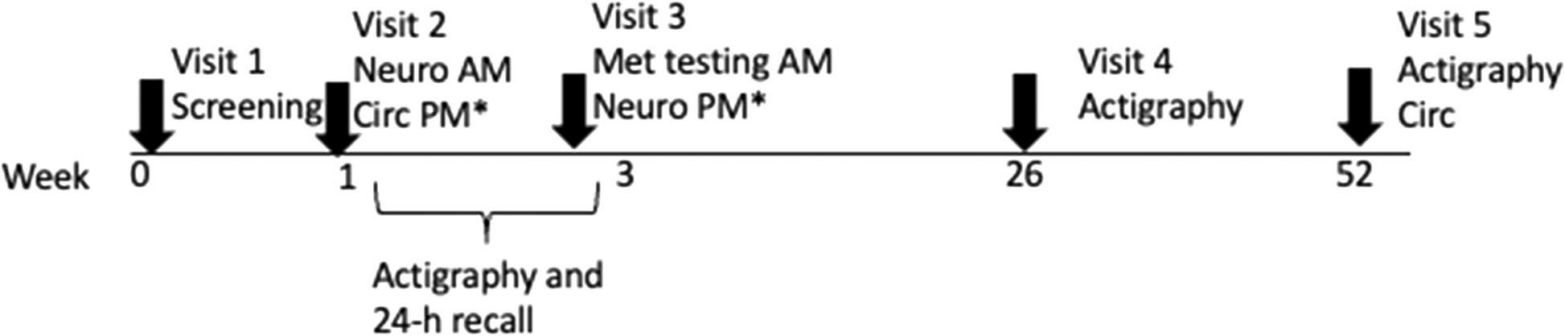
Procedure. * Indicates visits conducted in randomized counterbalanced order. Neuro= neurobehavioral testing, circ= circadian testing, met= metabolic testing

The study consists of 5 in-person visits (Fig. 1).

#### Visit 1

Screening/baseline. Participants who are potentially eligible based on the online or telephone prescreening will complete an in-lab screening (visit 1) which involves informed consent, blood draw for HbA1c, height and weight measurement to determine BMI criteria, one night with an Apnea Link monitor (Resmed Inc.) to screen for presence of OSA and 7 days of wrist actigraphy (brand, model) to screen for sleep timing criteria. After reviewing the screening data, research staff will call participants to inform them of their eligibility. Participants who are excluded due to high risk for OSA or diabetes will be provided with a results letter and recommended to discuss with a physician.

#### Visits 2 and 3

These visits will be scheduled in counterbalanced order (Day A and B), with approximately 14 days between sessions, to reduce carryover effects in the neurobehavioral testing. Day A starts at 9 am and consists of neurobehavioral testing in the morning session, questionnaires and free time mid-day (quiet laboratory activities such as watching TV or reading) and circadian phase assessment, (dim light melatonin onset, DLMO), in the evening. Participants will be discharged at their habitual bedtime. For Day B, participants arrive at the University of Utah Center for Clinical and Translational Science outpatient location at 8:30 am. Participants will be instructed to fast for the 8 h prior to their visit. Participants will undergo a frequently sampled IV glucose tolerance test from 9:00 am to approximately 12:30 pm. After this test, participants will go to the behavioral sleep medicine laboratory and will be provided with lunch. The afternoon will consist of completing self-report questionnaires and then participants will have free time for quiet activities in the laboratory. Snacks will be provided ad libitum. The neurobehavioral testing will start at 7:45 pm and discharge will occur around 9:30 pm.

#### Follow-up visits

Participants will complete wrist actigraphy, HbA1c, BMI and body fat assessments at 6 months (visit 4) and 12 months (visit 5). Visit 5 will also include a circadian phase assessment (DLMO).

Phone follow-ups: We will conduct follow-up surveys at 3 and 9 months to maintain engagement with the participants.

#### Procedure adaptations during COVID-19 Pandemic

In order to reduce risk for COVID exposure among participants and staff, we made the following adaptations in our procedure: We will minimize time in the room with the participant, for example instructing the participant to self-collect and store saliva samples with the use of a two-way video monitor, requiring KN95 or N95 facemasks by staff and participants at all times except when alone in a room with a closed door and finally, and offering the option for a limited contact at the 12-month follow-up visit: In order to reduce in-person exposure and preserve our primary outcome measures for visit 5 (HbA1c, body measurements collected in the lab, wrist actigraphy and questionnaires completed at home).

#### Compensation

Participants will be paid up to $850 for study participation if they complete all elements of the study. They will also receive additional compensation based on performance on the neurobehavioral tests (up to $30 per session).

### Measures

#### Screening measures

Participants will complete questionnaires to screen for insomnia symptoms using the Insomnia severity index [10], restless legs syndrome using the International Restless Legs Questionnaire [11], risk for obstructive sleep apnea using the STOP questionnaire [12], depression using the Patient Health Questionnaire-8 [12], substance alcohol use using the Alcohol Use Disorders Identification Test [13], drug use using the National Institutes on Drug Abuse (NIDA) Modified Assist questionnaire [14], and selected items to screen for symptoms of eating disorders (restriction, binging, purging) from the Eating Disorders Diagnostic Scale [15].

#### Wrist actigraphy protocol

Participants will wear the Actiwatch Spectrum (Philips/Respironics, Bend, OR) on the nondominant wrist for 7 days at screening/baseline, prior to the phase assessment, at 6 and 12 months. Actiwatches will be set with 30-s epoch length and medium sensitivity. Actigraphic sleep parameters will be calculated using Actiware-Sleep 6.1.1 software (or the current version) with default settings and included the following variables: sleep onset time, sleep offset time, and sleep duration. Actigraphy files will be scored using a standardized protocol using inputs of marker use, light and activity [16].

#### Circadian measures

Saliva samples will be collected every 30 min in the 7 h prior to habitual sleep onset time, as determined by actigraphy at screening. DLMO time will be calculated for each profile, and defined as clock time when salivary melatonin concentrations rise above the average of three low daytime values plus twice the standard deviation of the baseline values [17]. Circadian alignment will be calculated as duration between DLMO and average sleep onset time in adjacent week, consistent with our prior study [8].

#### Metabolic measures

We will assess HbA1c using the A1cNow Self Check test (PTS diagnostics). BMI and body fat will be measured using a Tanita scale. We will also collect waist and hip circumference measures. A frequently sampled intravenous glucose tolerance test (FSIGT) will be used to measure insulin sensitivity [18, 19]. Venous samples for plasma glucose and insulin will be drawn at -15, -10, -5, 3, 4, 5, 6, 8, 10, 14, 19, 22, 25, 27, 30, 40, 50, 60, 80, 100, 140, 180 min. Glucose (300 mg/kg) will be injected over 2 min at 0 min. Initially, our protocol stated that insulin (0.02 U/kg) would be infused at the 20-min time point, but the insulin was dropped from the protocol due to hypoglycemia in several of the initial participants. Minimal model analyses will be performed using the Minmod Millennium software [20]) which will provide the insulin sensitivity index (SI), the acute insulin response to glucose (AIRg) and the disposition index (DI = AIRg × SI), a marker of diabetes risk.

#### Neurobehavioral testing

The primary neurobehavioral measure will be the 5-trial adjusting delay discounting task [21]. We will also administer measures of inhibitory control (cite stop signal) [22], risk taking using the Balloon Analogue Risk Task (BART Task) [23] and processing speed (digit symbol) [24].

#### Eating behaviors

We will measure hedonic eating using the eating in the absence of hunger task [25], which consists of an oatmeal pre-load followed by a sham taste test. Eating behaviors in daily life will be measured using the Automated Self-Administered 24 h diet recall (ASA 24) [26]. Using values derived from the ASA 24, we will calculate the healthy eating index (HEI) as a composite measure of diet healthfulness [27].

Other self-report questionnaires administered include the PROMIS sleep disturbance measures [28] work hours and conditions [29] and questionnaires to measure perceived stress, demographics and household conditions [30–32].

### Data analysis

Data will be analyzed using R (Version 3.2)^61^ All analyses will be performed on an intent-to-treat basis. Significance will be defined p values < 0.05 on two tailed tests.

#### Aim 1

Cross-sectional relationships between circadian alignment, sleep duration and cardiometabolic health.

We will use a multivariable linear regression to model the relationship between insulin sensitivity index at baseline (the outcome) with the baseline circadian alignment, controlling for sex, age, and race/ethnicity. Secondary analyses will be conducted similarly using other measurements at baseline. Other variables, all taken at baseline, being modeled will include metabolic variables – acute glucose response, disposition index, HbA1c, BMI, and body fat – neurobehavioral variables – delay discounting and stop/signal – and behavioral variables – diet quality and physical activity.

In all models, transformation of the outcome will be done if needed for normality. Regression diagnostics will be examined to check for outliers or influential data points as well as for nonlinear relationships with circadian alignment. Additional exploratory analyses will add pairwise interaction terms to evaluate effect modification by sleep duration and sex.

#### Aim 2

We will examine the differences in morning and evening measurements in several outcomes. The primary outcome we will be examining is the calories consumed. Secondary outcomes will be neurobehavioral variables – delay discounting, stop/signal, and processing speed – and mood or hunger rating variables – sleepiness, hunger, and appetite. First, we will use paired t-tests to examine differences for all outcomes. We will then use multivariable linear regression to model the relationship between the difference (morning vs. evening) in calories consumed and DLMO time, controlling for sex, age, and race/ethnicity. Regression diagnostics will be examined for nonlinearity, outliers, and influential data points. For additional outcomes, we will examine similar models.

#### Aim 3

We will examine longitudinal changes in several outcomes over the course of a year, specifically at three time points – baseline, 6 months, and 12 months – and how they related to circadian alignment at baseline. We will first look at the relationship between our primary outcome, HbA1c, and circadian alignment (at baseline), while adjusting for covariates age at baseline, sex, race/ethnicity and sleep duration. Visit time (baseline, 6 months, and 12 months) will be modeled as a categorical factor and an interaction between visit time and our main predictor, baseline circadian alignment, as well as each of the covariates. We will use a linear mixed effects regression model with an unstructured covariance model to account for serial correlation. If convergence fails, we will compare alternative models for serial correlation, such as heteroscedastic auto-regressive models and compound symmetry, using likelihood tests or AICs to choose the preferred model. A similar procedure will be followed for additional outcomes BMI, body fat, diet and physical activity.

### Sample size justification

Sample size determinants: We aim to enroll 120 subjects and, accounting for a 20% attrition rate, this will allow us to collect complete data on 96 subjects. Based on standard deviations of 32.1 mg*L^2/(mmol*mU*min) or insulin sensitivity index [33] and 57 min for circadian alignment, we can estimate the minimum detectable effect at 80% power to be 0.161 mg*L^2/(mmol*mU*min) /min for a univariable linear regression. Further assuming a correlation of 0.4 between the covariates and circadian alignment, we can estimate the minimum detectable effect (of circadian alignment on insulin sensitivity index) in the multiple regression to be 0.176 mg*L^2/(mmol*mU*min). We will use all available resources to recruit the planned number of participants, but due to slowdowns and stoppages related to the COVID-19 pandemic, we may not reach the full sample size planned for this study. If we enroll 100 participants rather than 120, it will increase the minimal detectable effect size by 9.5%. If we enroll 110 participants, it will increase the minimal detectable effect size by 4.5%.

### Missing data

Our longitudinal analyses employ restricted maximum likelihood estimation which will provide approximately unbiased results in the presence of missing data so long as the pattern of missingness follows a missing at random pattern after accounting for the non-missing measurements of the variables included in the model [34]. Nonetheless, fully sequential multiple imputation will be the primary method used to address missing data [35]. The imputation models will include all variables in the respective outcome models plus additional variables identified as likely related to the risk of missingness or the values of the variables being imputed. In particular, any nonmissing baseline, 6 week or 12 week or 1-year measurements for a given outcome will be included in imputation models for that outcome. The application of multiple imputation will assure that statistical inferences are approximately unbiased so long as the mechanism of missingness follows a missing at random [MAR] structure [36] after accounting for both the analysis variables and additional auxiliary variables in the imputation models.

### Data collection and management

Data will be captured using the REDCap automated data system. Measures will be directly entered into the case report forms whenever possible. We will score and process wrist actigraphy files quarterly. Dr. Baron will visually review scoring for all files and re-score at least 10% of scored wrist actigraphy quarterly for quality control. If values are > 10% different, the study coordinator will undergo re-training.

## Discussion

This study extends results of laboratory-based experimental research to evaluate the impact of these sleep/circadian factors on adults in their daily lives. Results will advance understanding of the mechanisms by which sleep and circadian rhythms link to the neurobehavioral processes that affect eating behaviors, including impulsivity and the underlying metabolic risk factors. We will be able to examine how time of day affects the ability to delay gratification and hedonic eating behaviors and also whether sleep and circadian alignment affect change in cardiometabolic risk over 1-year period.

Strengths of this study include detailed, objective measurement of sleep and circadian timing, gold-standard methods of metabolic testing, and use of standardized neurobehavioral tests and ecologically valid tests of hedonic eating behaviors. On the other hand, limitations to this study include a highly screened sample free from depression, sleep disorders and substance abuse (which limits generalizability). The ongoing effects of the COVID-19 pandemic on enrollment and retention on research studies may affect the power to observe our hypothesized relationships, despite our best efforts to overcome these challenges.

In conclusion, this protocol will examine key neurobehavioral and metabolic processes that are hypothesized to link sleep loss and circadian rhythm misalignment to increased risk for cardiometabolic disorders. Results will advance the translation of laboratory research by increasing the understanding of how circadian rhythm misalignment and short sleep duration interact to increase risk for cardiometabolic diseases in naturalistic settings.

## Data Availability

N/A.

## Abbreviations

BMI: Body mass index
DLMO: Dim light melatonin onset
HbA1c: Glycated hemoglobin
SI: Insulin sensitivity index
AIRg: Acute insulin response to glucose
DI: Disposition index
BART: Balloon analogue risk task
ASA-24: Automated self-administered 24 h diet recall
HEI: Healthy eating index
